# Preventive Effects of Tri Garn Pis Polyherbal Extract on Sexual Performance, Testicular Apoptosis, and Sperm Quality in a Dexamethasone-Induced Chronic Stress in Mice

**DOI:** 10.3390/life16010116

**Published:** 2026-01-13

**Authors:** Chadaporn Chaimontri, Sitthichai Iamsaard, Tarinee Sawatpanich, Nongnut Uabundit, Arada Chaiyamoon, Rarinthorn Samrid, Therachon Kamollerd, Chayakorn Taoto, Natthapol Lapyuneyong, Sararat Innoi, Tidarat Chawalchitiporn, Pornpan Kerdsang, Nawaphon Koedbua, Yutthaphong Patjorn, Chanasorn Poodendaen, Suthat Duangchit, Supatcharee Arun

**Affiliations:** 1 Department of Anatomy, Faculty of Medicine, Khon Kaen University, Khon Kaen 40002, Thailand; chadaporn_chaimontri@kkumail.com (C.C.); sittia@kku.ac.th (S.I.); tarinee@kku.ac.th (T.S.); nongua@kku.ac.th (N.U.); aradch@kku.ac.th (A.C.); rarisa@kku.ac.th (R.S.); therachon_k@kkumail.com (T.K.); chayakorntaoto@kkumail.com (C.T.); l.natthapol@kkumail.com (N.L.); sararat.i@kkumail.com (S.I.); tidarat.ch@kkumail.com (T.C.); pornpan.ke@kkumail.com (P.K.); nawaphon.ko@kkumail.com (N.K.); yutthaphong.p@kkumail.com (Y.P.); 2 Department of Anatomy, Faculty of Medical Science, Naresuan University, Phitsanulok 65000, Thailand; chanasornp@nu.ac.th; 3 Department of Physiology, Faculty of Medical Science, Naresuan University, Phitsanulok 65000, Thailand; suthatd@nu.ac.th

**Keywords:** Tri Garn Pis recipe extract, dexamethasone, sexual behavior, sperm motility, testis

## Abstract

Chronic stress (CS) contributes to male infertility, reduced testosterone levels, and impaired semen quality. CS models induced by glucocorticoids, such as dexamethasone (DEX), negatively affect sperm parameters and testicular health, notably by promoting testicular apoptosis. While individual plant extracts have been studied for their ability to mitigate stress-induced reproductive dysfunction, the preventive effect of the Tri Garn Pis (TGP) polyherbal extract in DEX-induced CS (DexCS) has not previously been investigated. This study evaluated the effects of TGP extract on testicular function, sexual behavior, and sperm quality in DexCS male mice. Seventy-two ICR mice were randomly divided into six groups: control, DexCS, TGP (50, 100, and 200) + DexCS, and TGP200. Mice received TGP (50, 100, 200 mg/kgBW) for 14 days before DEX co-treatment for 28 days. Behavioral and reproductive assessments included depression-like behavior tests, sexual behavior, sperm quality, testicular histopathology, steroidogenesis proteins (AR, CYP11A1, StAR), and apoptosis markers (Hsp70, caspase-3, caspase-9). TGP extract—which is rich in phenolics and flavonoids with antioxidant activity—improved depressive behavior, sexual performance, testicular histology, and low sperm quality. TGP also upregulated testicular StAR expression while reducing caspase-3 and caspase-9 levels. TGP prevents testicular apoptosis, sexual dysfunction, and poor sperm motility induced by DexCS.

## 1. Introduction

Chronic stress (CS) is increasingly recognized as a significant risk factor for numerous systemic diseases, particularly cardiovascular and gastrointestinal disorders, as well as reproductive dysfunctions such as male infertility [1,2]. The underlying mechanism involves excess activation of the hypothalamic–pituitary–adrenal (HPA) axis, resulting in elevated secretion of corticotropin-releasing hormone, adrenocorticotropin hormone, and cortisol. In humans, CS has been shown to cause erectile dysfunction, reduced libido, and decreased sex hormone levels, leading to poor semen quality and male reproductive potential [3]. CS is a major factor in male reproductive dysfunction in rodents, as evidenced by reductions in sexual behavior, spermatogenesis, and increased testicular apoptosis [4]. Mechanistically, stress directly impaired testicular function by reducing expressions of key steroidogenic proteins, including steroidogenic acute regulatory protein (StAR) and cytochrome P450 family 11 subfamily A member 1 (CYP11A1), leading to low testosterone levels and sperm quality [4,5,6,7].

Exposure to corticosteroids such as dexamethasone (DEX), which is widely used to induce stress-related reproductive impairments in animal models, stimulates the deleterious effects observed in human stress-associated infertility and sexual dysfunction [8,9]. Chronic DEX administration has been widely used to mimic stress in animal models and depressive-like behavior in rodents, with established protocols demonstrating its effects in both mice and rats [10,11,12]. In dexamethasone-induced chronic stress (DexCS) models, DEX injection for 21 consecutive days induces behavioral and physiological changes characteristic of depression and stress-related disorders [12,13,14]. Importantly, DEX exposure has been shown to decrease the body weight of mice [12,13,15,16]. Furthermore, DexCS significantly reduces testosterone levels, which correlate with low testicular steroidogenic acute regulatory protein (StAR) immunoreactivity, seminiferous epithelium, and poor sperm quality [17,18,19,20,21]. A recent study also reported significant upregulation of testicular cleaved caspases-3 and -9 in DexCS groups compared to controls, thereby enhancing apoptotic signaling under chronic DEX exposure [12]. Moreover, evidence from environmental toxicology shows that low-level exposure to chromium can disrupt sperm nuclear proteins and increase susceptibility to oxidative DNA damage [22]. More recent studies demonstrate that environmental pollutants impair semen quality through oxidative stress, disturbed trace metal balance, and changes in epigenetic and nuclear packaging markers consisting of protamine, small noncoding RNAs, even when routine spermiograms appear normal [23].

Drugs have recently been used in clinical settings to treat hypogonadism-induced male infertility, including hormonal medications such as clomiphene anastrozole, letrozole, and exemestane [24]; antioxidants like vitamin E and astaxanthin [25]; and traditional herbal medicines [4,26,27]. However, the use of exogenous hormone therapies for male infertility is limited due to their adverse effects, including changes in libido, mood disturbances, headaches, nausea, and potential impacts on bone mineral density [28]. In traditional medicine, several synergists of plant extracts, such as tri formula, have gained increasing attention for treating many diseases. The Tri Garn Pis (TGP) recipe is a tri formula, containing three major Thai medicinal herbs (red basil roots [*Ocimum tenuiflorum* L.], kha ling [*Alpinia conchigera* Griff.], and fingerroot rhizomes [*Boesenbergia rotunda* L. Mansf.]), as described in previous studies [29,30]. Traditional medicine practitioners believed that the TGP recipe possesses properties for treating nourishment, carminative, league, blood circulation, and anti-fungal activity, especially aphrodisiac activity [29,30].

Recent research has demonstrated that the single extracts from plants of the same family as TGP, such as *O. tenuiflorum* L. roots, *B. rotunda* (L.) Mansf. rhizome, and *A. galanga* (L.) Willd., and the Tri-kanpis remedy contain a range of phytochemicals, including anthraquinones, cardiac glycosides, steroids, and terpenoids [31]. To support the aphrodisiac activity of individual plants, a previous study reported that aglycones in *O. tenuiflorum* L. root could increase testosterone levels [32]. Bioactive constituents of the *A. conchigera* Griff. rhizome, including β-sitosterol, flavonoids, and 1′S-1′-acetoxychavicol acetate, attenuate seminiferous tubular abnormality and low sperm quality [33,34,35]. Additionally, quercetin and kaempferol, found in *B. rotunda* (L.) Mansf. Rhizome, exhibit antioxidant activities that protect against testicular damage and enhance male sexual behaviors, including mounting, intromission, and ejaculation [35,36,37,38,39]. In addition, extracts from the leaves and rhizomes of *O. tenuiflorum* L., *Ocimum sanctum* L., and *A. conchigera* Griff. rhizomes exhibit anticancer properties by upregulating apoptotic genes (BAX and BAK) and apoptotic markers (caspase-3 and caspase-9) while downregulating anti-apoptotic genes (BCL-2 and BCL-xL) [40,41,42]. Extracts from *Boesenbergia rotunda* protect kidney function by reducing key indicators of kidney damage (Kim-1 and NGAL), suppressing inflammation-related genes (NF-κB), and decreasing cell death genes (caspase-3 and caspase-7) [43]. Moreover, Wuzi Yanzong Pills (WZYZ Pills)—a traditional Chinese medicine formula—could ameliorate testicular inflammation by decreasing inflammatory factors, including IL-6, TNF-α, and IFN-β, thereby improving seminiferous tubule atrophy and sperm quality in rats induced by testicular heat stress [44]. Although the effects of single plant extracts on reproductive dysfunctions under chronic stress have been revealed in previous studies, the preventive effects of Tri Garn Pis (TGP) recipe extract on sexual performance and male reproductive function in DEX-induced mice have not been reported. We hypothesize that TGP exerts protective effects against DEX-induced reproductive dysfunction through antioxidant activity and by preserving testicular steroidogenic proteins (e.g., StAR, CYP11A1) and androgen receptor signaling while reducing activation of apoptotic pathways. Therefore, this study aims to systematically evaluate the efficiency of Tri Garn Pis recipe extract on aphrodisiac activity and testicular function, including sperm quality in male mice exposed to DexCS.

## 2. Materials and Methods

### 2.1. Plant Collection and Authentication

Three plant components—red basil roots (*Ocimum tenuiflorum* L.), kha ling (*Alpinia conchigera* Griff.), and fingerroot rhizomes (*Boesenbergia rotunda* (L.) Mansf.)—were collected and used as an ingredient for preparing the Tri Garn Pis (TGP) recipe. 1. Red basil seeds were planted at Mueang Phia Subdistrict, Kutchap District, Udon Thani Province, Thailand. At plant maturity, the leaves and stems turned from green to reddish-purple, indicating full growth and the presence of all essential components. Fresh red basil, including stems, leaves, flowers, branches, and roots, was then collected by Miss Chadaporn Chaimontri, Department of Anatomy, Faculty of Medicine, Khon Kaen University. 2. Fresh kha ling and fresh fingerroot (including rhizomes, flowers, and leaves) were obtained from a wild plant gatherer who lives in Hin Hao Subdistrict, Lom Kao District, Phetchabun Province, Thailand.

**Figure 1:**
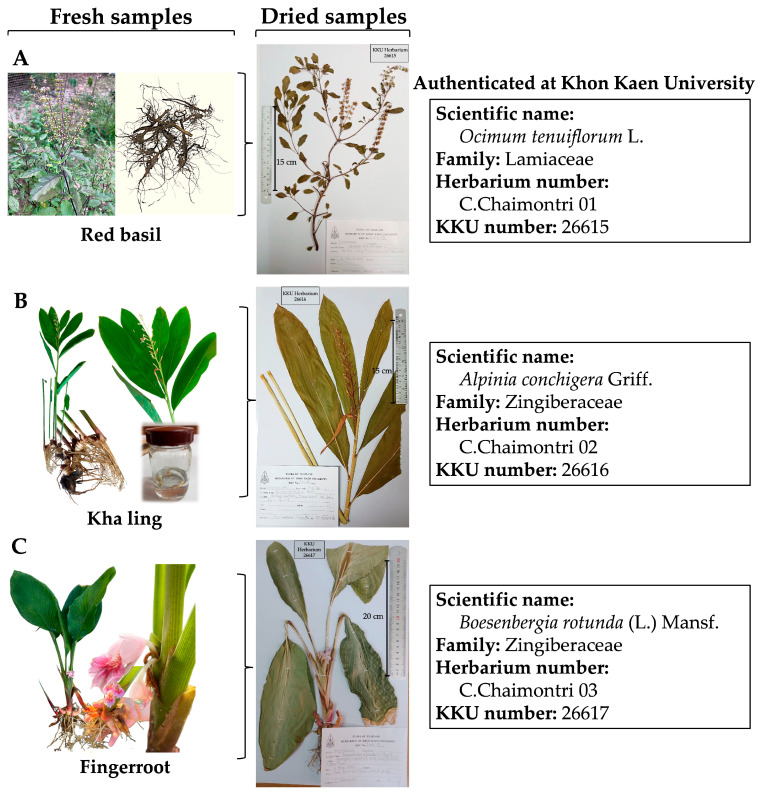
Preparation of voucher specimen and identification of *Ocimum tenuiflorum* L. tree (**A**), *Alpinia conchigera* Griff. (**B**), and *Boesenbergia rotunda* (L.) Mansf. (**C**).

For identification and preparation of voucher specimens, all collected plant samples were sent to the Department of Biology, Faculty of Science, Khon Kaen University, Thailand, and were authenticated by Dr. Pornchai Kladwong. Subsequently, fresh fingerroot samples were transferred and arranged into a herbarium cassette (30 cm in width × 45 cm in length) and dried (at 40–60 °C) for one week. Lastly, the species of dried plant specimens were identified and authenticated as *Ocimum tenuiflorum* L. (Lamiaceae family), *Alpinia conchigera* Griff., and *Boesenbergia rotunda* (L.) Mansf. (Zingiberaceae family). Voucher specimens were deposited in a KKU herbarium (number of C. Chaimontri 01-03, KKU number is 22615-26617) as shown in Figure 1.

### 2.2. Tri Garn Pis (TGP) Recipe Preparation and Extraction

Individual plant samples were collected, washed, and air-dried for 3–4 days. Each plant sample was sliced into small pieces using a kitchen blade and weighed before extraction, which was performed twice. For the first extraction, all small pieces of the three plants were mixed (1:1:1) to prepare the Tri Garn Pis (TGP) recipe, with a total weight of 2550 g. Then, the mixed plants were immersed in 12.75 L of 50% ethanol (mixed plant [1 g]: 50% Eth [5 mL] ratio) in the hermetically sealed container for 7 consecutive days at room temperature. To increase extraction yields, the TGP extract, immersed in ethanol, was continually stirred by using a ladle twice daily (morning [9:00 a.m.] and afternoon [4:00 p.m.]) for 60 s each time. After 7 days, the mixture was filtered through nylon cloth to separate the ethanol extract (TGP extract) from the plant residues.

For the second extraction, the TGP residues after the first extraction were mixed with 50% ethanol at the same ratio. This second extract was also filtered through nylon cloth.

**Figure 2:**
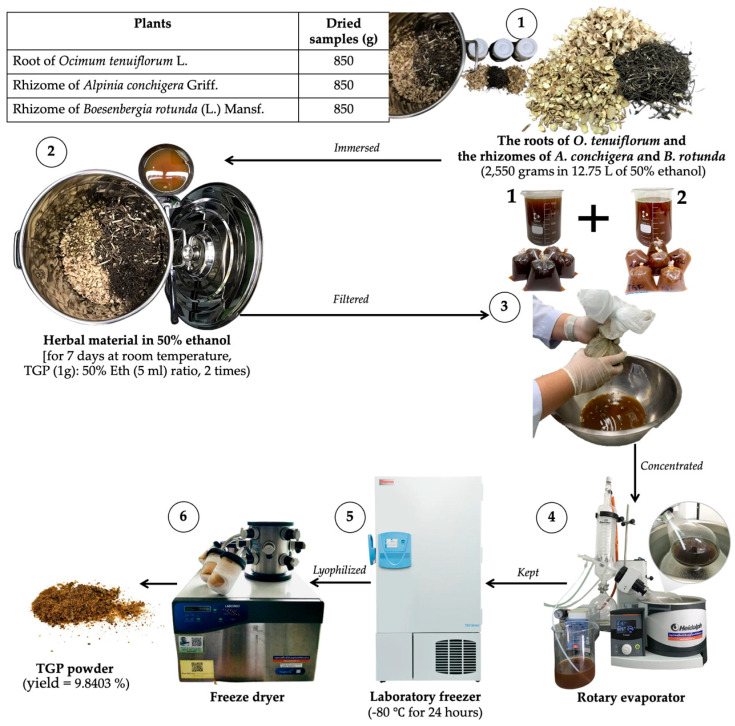
The process of plant ethanol extraction, filtration, evaporation, and lyophilization of Tri Garn Pis (TGP) recipe.

The first and second extracts were then combined and stored at −80 °C for 24 h before undergoing evaporation and lyophilization at the Faculty of Medicine, Khon Kaen University, Thailand. The resulting lyophilized powder was caramel-like in color, as shown in Figure 2. Finally, the crude powder extract was calculated as a percentage yield (9.8403%).

### 2.3. Evaluation of Total Phenolic Content and Flavonoid Compound of TGP Recipe Extract

The modified colorimetric Folin−Ciocalteu method previously described in [45] was used to determine the amounts of phenolic contents in TGP extract. This assay is based on the ability of TGP to reduce the molybdenum (Mo^6+^ to Mo^5+^) and tungsten (W^6+^ to W^5+^) ions in the phosphomolybdic and phosphotungstic complex reagents, leading to a color change from yellow to blue due to electron transfer from the phenolate anions to molybdenum. The distilled water (DW, 500 μL) and diluted extract (conc. at 0.01 mg/mL, 125 μL) were added to a test tube. Folin−Ciocalteu reagent (125 μL) was added to the solution and allowed to react for 6 min. Then, 7% sodium carbonate solution (1250 μL) was added to the test tube, and the mixture was diluted with DW (1000 μL). After incubation for 90 min at room temperature, the absorbance was read at 760 nm in triplicate. The measurement was compared to a standard curve plotted using the gallic acid solutions (30, 60, 90, 120, 150, 180, 210, 240 μg/mL). The total phenolic contents of the TGP extract were reported as mg GAE (gallic acid equivalent)/g TGP.

The total flavonoid content in TGP extract was evaluated using aluminum chloride (AlCl_3_) colorimetric assay as previously described [45]. This assay is based on the formation of a stable yellow complex between aluminum chloride and specific functional groups in flavonoid compounds, particularly the keto group at the C-4 position and the hydroxy groups at the C-3 or C-5 positions in flavones and flavonols. Briefly, the TGP extract (conc. at 0.01 mg/mL, 125 μL) was mixed with DW (1250 μL) before adding 5% sodium nitrite solution (75 μL); the mixture was incubated for 5 min. Then, 150 μL of 10% aluminum chloride solution was added and incubated for 6 min, followed by the addition of 500 μL of 1 M sodium hydroxide. After that, the solution was added with DW (275 μL), and the absorbance was read at 510 nm in triplicate. The calibration curve of TGP extract was plotted by using catechin as a standard (30, 60, 90, 120, 150, 180, 210, 240, 270, and 300 μg/mL). The concentrations of total flavonoid content were expressed as milligrams of catechin per gram TGP extract (Supplementary File S1).

### 2.4. Scavenging Effect on DPPH Free Radical

To determine the radical-scavenging activity of TGP extract, the 2,2-Diphenyl-1-picrylhydrazyl (DPPH) radical-scavenging assay was performed following a previously described protocol [46]. The radical scavenging ability of TGP extract (at five concentrations) was compared to that of the standard compounds, including butylated hydroxytoluene (BHT), alpha (α)-tocopherol, and ascorbic acid. Briefly, TGP extract (1 mL) was mixed with 0.1 mM of DPPH in 95% ethanol (1 mL) and incubated in a dark room for 30 min at 25 °C. Then, the absorbance was measured at 517 nm in triplicate. The percentage of DPPH radical scavenging activity was calculated using the formula: DPPH radical scavenging activity (%) = [(A_0_ – A_1_)/A_0_] × 100, where A_0_ = absorbance of control and A_1_ = absorbance of sample. In addition, radical scavenging activity (%) at various concentrations of TGP was calculated by a half maximal inhibitory concentration (IC_50_) of the standard compound. Detailed conditions are provided in Supplementary File S1.

### 2.5. Ferric Reducing/Antioxidant Power (FRAP) Assay

Antioxidant capacity was determined by using the ferric reducing antioxidant power (FRAP) assay, as previously described [47]. First, 0.1 mL of TGP extract solution (1 mg/mL) was incubated with FRAP reagent containing 300 mM/L of acetate buffer, 20 μM/L of FeCl_3_·6H_2_O, 10 μM/L of TPTZ (2,4,6-tris(2-pyridyl)-s-triazine) dissolved in HCl (40 μmol), and DW. The mixture was further incubated at 37 °C for 4 min, after which the absorbance was measured at 593 nm in triplicate using an ultraviolet–visible spectrophotometer. The reducing power capacity of TGP extract was compared to a standard curve (FeSO_4_·7H_2_O: 0.1, 0.2, 0.4, 0.6, 0.8, and 1 mM) and plotted from various concentrations of BHT, α-tocopherol, and ascorbic acid; results are reported as μmol of Fe (II)/1 g TGP as shown in Supplementary File S1.

### 2.6. Identification of Active Compounds by Using HPLC

Active compounds in the TGP recipe extract were identified and quantified using high-performance liquid chromatography (HPLC; Shimadzu Nexera LC-40 series, Shimadzu Co., Kyoto, Japan) at the Kasetsart Agricultural and Agro-Industrial Product Improvement Institute, Kasetsart University, Bangkok, Thailand. Sample preparation involved dissolving 0.2 g of TGP extract in 25 mL of absolute methanol, followed by filtration through a 0.45 μm membrane filter before injection. Compound identification was performed using the external standard method by comparing retention times and UV spectra with available authentic standards: ascorbic acid, gallic acid, caffeic acid, chlorogenic acid, hesperidin, and curcumin. Two chromatographic methods were employed: (1) For ascorbic acid, gallic acid, caffeic acid, hesperidin, and chlorogenic acid, separation was achieved on an InertSustain C18 column (4.6 mm ID × 250 mm, 5 μm; GL Science, Torrance, CA, USA) using gradient elution of 0.1% acetic acid and acetonitrile at 0.6–1.0 mL/min. Detection wavelengths were optimized for each compound: 278 nm (ascorbic acid), 272 nm (gallic and caffeic acids), 283 nm (hesperidin), and 325 nm (chlorogenic acid). (2) Curcumin analysis utilized an Inertsil ODS-4 column (3.0 mm × 150 mm, 5 μm; GL Science, Torrance, CA, USA) with water–acetonitrile gradient elution at 0.5 mL/min and detection at 425 nm. Quantification was performed using external calibration curves prepared from 5 to 7 concentration points: ascorbic acid (5–80 μg/mL), gallic acid, caffeic acid, and hesperidin (3.125–50 μg/mL), chlorogenic acid (3.125–100 μg/mL), and curcumin (3.125–50 μg/mL) (Supplementary File S2).

### 2.7. Animals and Experimental Design

Seventy-two male and thirty-six female ICR mice (35–40 g, 10 weeks old) were purchased from the Northeast Laboratory Animal Center, Khon Kaen University, Thailand. All animals were housed in plastic cages under the experimental room condition (12 h light/dark cycle, temperature of 23 ± 2 °C, humidity of 30–60% RH, sound level below 85 decibels, and light intensity of 350–400 lux) in Northeast Laboratory Animal Center, Khon Kaen University, Khon Kaen, Thailand. Then, they were provided with commercial pellet food and water ad libitum. All mice were acclimatized for 7 days. This study was approved by the Institutional Animal Care and Use Committee of Khon Kaen University, based on the Ethics of Animal Experimentation of Nation Research Council of Thailand (recorded code number: IACUC-KKU-66/66).

After acclimatization for 7 days, the experiment was separated into two phases: preventive and co-treatment. Baseline data were collected by conducting sexual behavior (training and test), tail suspension, and forced swimming tests. These tests were performed before and after treatment to confirm chronic stress regarding related behaviors and to compare among groups. Animals were randomly divided into 6 groups (12 mice/each group): control, dexamethasone-induced chronic stress (DexCS), TGP 50 plus DexCS, TGP 100 plus DexCS, TGP 200 plus DexCS, and TGP 200 alone. In the co-treatment period, mice were intraperitoneally injected with 4 mg/kgBW dexamethasone (DEX) for 28 consecutive days to reliably induce chronic-stress-like behaviors and reproductive dysfunction in experimental animals [13,14]. During the same period, chronic stress in mice was evaluated by the sucrose preference test. For the preventive period, animals in the control and DexCS groups received 1% of dimethyl sulfoxide (DMSO), while those in other groups received TGP extract dissolved in 1% of DMSO at doses of 50, 100, or 200 mg/kgBW via oral gavage. For the co-treatment period, control mice were not subjected to chronic stress induction, but were intraperitoneally injected with 3 mmol/mL of sodium phosphate (Na_3_PO_4_), the solvent for dissolving DEX. Mice in the DexCS group were injected with DEX (i.p.). For the TGP extract plus DexCS groups, mice were gavaged with TGP at doses of 50, 100, or 200 mg/kgBW based on previous studies in rodents [48,49,50]. After 1 h of TGP extract treatment via oral gavage, mice were injected with DEX at 4 mg/kg. In the last group, all mice were gavaged with a high dose (200 mg/kgBW) of TGP in the preventive and co-treatment periods to evaluate the potential toxicity of the TGP extract on reproductive organs. At the end of the experiment, all animals were sacrificed.

### 2.8. Assessments of Chronic Stress

The sucrose preference, tail suspension, and forced swimming tests were used to evaluate chronic stress behaviors in mice involved with DexCS as previously described [12].

### 2.9. Sexual Behavior Test

All sexual tests were conducted in a quiet, dimly lit room. For acclimatization, each male mouse was placed in the center of a plexiglass box (24 cm × 35 cm × 20 cm), a transparent acrylic observation box, for 10 min. To gain estrous animals, thirty-six adult female mice (12 weeks) were subcutaneously injected with estradiol benzoate (10 mg/mouse; Sigma-Aldrich) at 48 h and progesterone (500 mg/mouse; Sigma-Aldrich, St. Louis, MO, USA) at 4 h before testing [51]. Then, a female mouse with an estrus phase—confirmed based on the presence of cornified epithelial cells from vaginal smear—was gently placed into the behavioral testing arena with a male mouse. Next, the mating behaviors, including frequency and latency of mounting, intromissions, ejaculations, and post-ejaculation interval (PEI), were observed and recorded for 30 min using a video camera (Logitech^®^ HD Webcam C270, Newark, NJ, USA) linked to computer recording software ver.29.1.1 (OBS studio program, Free Software Foundation, Inc., Boston, MA, USA). Both male and female mice were taken out of the testing box and placed back in their own home cage. The apparatus was cleaned with 75% ethanol before the next test.

The sexual behaviors in this study were previously described [52,53] as follows: the number of mounts until first ejaculation (mount frequency, MF), the time interval from introducing the female mouse to the first mount (mount latency, ML), the number of intromissions until first ejaculation (intromission frequency, IF), the time interval from introducing the female mouse to the first intromission (intromission latency, IL), the number of ejaculations observed within a series of sexual behavior test (ejaculation frequency, EF), the time interval from first intromission to ejaculation (ejaculation latency, EL), and the time from ejaculation to next intromission (post-ejaculation interval, PEI).

### 2.10. Reproductive Organ Collections

At the end of the experiment, mice were anesthetized by intraperitoneally injecting 80 mg/kgBW of thiopental sodium before being euthanized by cervical dislocation. The testis, epididymis plus vas deferens, penis, and seminal vesicle plus prostate gland were collected and weighed. The weights of body and reproductive organs were calculated and expressed as the relative weights (100 × absolute organ weight divided by final body weight). The seminal vesicle plus prostate gland, penis, and right reproductive organs (testis and epididymis plus vas deferens) were fixed with 10% formalin fixative solution for further histological study. The left testis of each mouse was snap-frozen to maintain freshness until further investigation of functional protein and apoptotic protein expressions.

### 2.11. Sperm Analyses

The left caudal epididymis plus deferens were removed to collect sperm for quality evaluations, including motility, concentration, morphology, viability, and acrosome status. Briefly, the sperm masses from the caudal epididymis were squeezed and suspended in normal saline (37 °C). Then, the sperm suspension was aliquoted to analyze the following sperm parameters.

#### 2.11.1. Sperm Motility, Concentration, and Sperm Abnormality

The sperm progressive motility, concentration, and sperm abnormalities were analyzed using computer-assisted sperm analysis (CASA) (IVOS^®^ II, Hamilton Thorne Inc., Beverly, MA, USA) as previously described [12]. The loaded chamber (20 μm in depth) was placed on the thermal plate of the microscope (37 °C) and heating plate (37 °C) before analysis. Briefly, the sperm suspension (6 μL) was loaded into a two-chamber slide (Leja Products B.V., Nieuw-Vennep, Netherlands). Sperm movement and non-movement were observed under negative phase contrast and recorded as videos for six fields per chamber. Finally, the results of sperm motility (progressive), total sperm concentration, and sperm abnormality (bent tail and proximal droplet) are reported as million/mL and percentage (%).

#### 2.11.2. Sperm Viability

The sperm viability was evaluated using eosin–nigrosin staining (1:1 ratio; eosin: Merck KGaA, Darmstadt, Germany; nigrosine: Cat. No. 72470, Fluka, Darmstadt, Germany) as previously described [12,27]. Sperm suspension (40 μL) was mixed with eosin–nigrosin dyes (40 μL) and incubated at 37 °C for 30 sec. Then, the stained sperm drop was smeared on a glass slide to observe the viability of sperm under a light microscope: alive sperm (non-stained) and dead sperm (pink). A total of two hundred sperm from each mouse were counted, and the percentage of viable sperm was calculated.

### 2.12. Sperm Acrosome Integrity Using Peanut Agglutinin (PNA) Lectin Staining

To differentiate between acrosome-intact and acrosome-reacted sperm, the fluorescein isothiocyanate (FITC)-labeled peanut agglutinin (PNA) lectin was used as a marker for monitoring the sperm acrosome reaction [54]. A total of 10 ul of fixed sperm in 4% paraformaldehyde was smeared on glass slides and dried overnight. For the heat-induced retrieval, the slides were incubated in 1× PBS buffer and subjected to microwave heating (560 W) for 3 times of 5 min each, followed by cooling to room temperature and PBS washes. Non-specific binding was blocked in 1% BSA in 1× PBS for 15 min. The smear slides were then incubated in the dark with Alexa Fluor™ 488-conjugated Peanut Agglutinin (PNA, 1:300, Cat. No. L21409, Thermo Fisher Scientific Inc., Waltham, MA, USA) for 45 min at room temperature, counterstained, and mounted with VECTASHIELD^®^ containing DAPI (4′,6-diamidino-2-phenylindole, Cat. No. H-1200-10, Vector Laboratories, USA). The lectin specifically binds to terminal β-galactose residues found in the acrosomal region. The 200 sperm per sample were observed and counted under a Nikon Eclipse Ni LED fluorescent microscope (Nikon, Tokyo, Japan). Acrosome-intact sperm exhibited intense green fluorescence, while acrosome-reacted sperm showed weak, irregular, or absent staining. The percentage of acrosome-reacted sperm was calculated as described previously [12,26].

### 2.13. Histological Analysis

The right testis and caudal epididymis, penis, and seminal vesicle were fixed with 10% formalin in DW (pH 7.4) for 48 h before paraffin tissue processing. The paraffinized tissue blocks were sectioned (thickness 5–7 μm) using an automatic microtome (ERM 3100, Hestion, Melbourne, Australia). The sections were deparaffinized, rehydrated, hydrated, and routinely stained with hematoxylin and eosin dyes. All histopathologies were observed under a light microscope (ZEISS Axio Imager.A2, Jena, Germany) and representative images were captured with an AxioCam ICc 5 digital camera (Carl Zeiss Microscopy GmbH, Jena, Germany). For quantitative analysis, the diameter and height of seminiferous tubule testis of such epithelium layer were measured on four transverse axes and quantified using the ImageJ program (Version 1.50i, National Institutes of Health, Bethesda, MD, USA).

### 2.14. Masson’s Trichrome Staining

The tissue sections were deparaffinized with xylene and rehydrated with serial descending alcohols. Then, the sections were immersed in Bouin’s solution and stained with Weigert’s iron hematoxylin. Subsequently, the sections were stained with Masson’s trichrome staining kits (Catalog no. HT15, Sigma-Aldrich, Inc., St. Louis, MO, USA). Finally, the sections were washed with 1% acetic acid. Histological changes in vital and reproductive organs were observed and photographed using a light microscope (ZEISS Axio Imager.A2, Germany) and an AxioCam ICc 5 digital camera (ZEISS, Germany) [55].

### 2.15. Western Blotting Analysis

Total testicular proteins were extracted using homogenization in 1× radioimmuno-precipitation assay (RIPA) buffer (Cell Signaling Technology, Inc., Danvers, MA, USA USA) containing protease inhibitor cocktails (EDTA-Free,100× in DMSO, MedChemExpress (MCE), NJ, USA). The lysate was then sonicated (20 Hz, 60 pulses) with the ultrasonic processor (Cole, Parmer, Vernon Hills, IL, USA) and centrifuged at 12,000 rpm, 4 °C for 10 min to separate soluble total proteins from the pellet. Total protein concentration was measured at an absorbance of 280 nm using a spectrophotometer (NanoDrop ND 1000 Technologies, Inc., Wilmington, DE, USA). Then, total testicular proteins (100 μg) were separated on 10% SDS PAGE (sodium dodecyl-sulfate on a 10% polyacrylamide gel electrophoresis) before blotting onto nitrocellulose membranes (Cat. No. 10600004, GE Healthcare Life Science, Chicago, IL, USA). Briefly, the non-specific binding was blocked with 5% bovine serum albumin (BSA) in 0.1% TBST overnight. Subsequently, membranes were washed and incubated with primary antibodies against androgen receptor (AR; Cat. No.06-680, Merck Millipore Co., Billerica, MA, USA), heat shock protein 70 (Hsp70; Cat. No. MAB3516, Abcam, Cambridge, UK), cytochrome P450 family 11 subfamily A member 1 (CYP11A1; Cat. No. sc-18040, Santa Cruz Biotecnology Inc., Dallas, TX, USA), steroidogenic acute regulatory (StAR; Cat. No. sc-25806, Santa Cruz Biotecnology Inc., Dallas, TX, USA), tyrosine phosphorylated (TyrPho; Cat. No. 05-321, Merck Millipore Co., Billerica, MA, USA), caspase 3 (Cat. No. sc-7272, Santa Cruz Biotecnology Inc., Dallas, TX, USA), caspase-9 (Cat. No. sc-56076, Santa Cruz Biotecnology Inc., Dallas, TX, USA), and glyceraldehyde-3-phosphate dehydrogenase (GAPDH; Cat. No. ab8245, Cambridge, Abcam, UK) at 4 °C for overnight. Then, each membrane was incubated with a specific secondary antibody conjugated with horseradish peroxidase (HRP). Finally, the enhanced chemiluminescence (ECL) substrate kit (GE Healthcare Life Science, Chicago, IL, USA) was used to detect specific Ab-Ag complexes on the membrane, visualized under Gel Documentation 4 (ImageQuant 600 GE Healthcare, Chicago, IL, USA). The relative intensity of testicular and sperm protein expressions was quantified using the ImageJ program (Version 1.50i, National Institutes of Health, Bethesda, MD, USA), with anti-GAPDH as an internal control.

### 2.16. Statistical Analysis

All data are expressed as mean ± standard deviation (SD). The one-way analysis of variance (ANOVA) and Bonferroni post hoc test were used to compare the differences among groups. The statistical analyses were carried out using GraphPad Prism 10 (GraphPad Software, Inc., Boston, MA, USA). A *p*-value of < 0.05 was considered statistically significant.

## 3. Results

### 3.1. Potential Antioxidant Contents and Activities of Tri Garn Pis (TGP) Recipe Extract

In the ethanolic TGP extraction, the yield of TGP powder extract was approximately 9.8403%. The total phenolic and flavonoid contents of the TGP extract, calculated from the gallic acid standard curve equivalent and catechin calibration curve, respectively, were 15.86 ± 0.27 mg GAE/g TGP extract and 6.68 ± 0.17 mg catechin/g TGP extract (Table 1). The IC_50_ of this powder extract was determined to be 644.87 ± 2.73 mg/mL via the DPPH assays. Moreover, the antioxidant activity of TGP, examined by FRAP value, was 151.25 ± 0.13, as shown in Table 1.

### 3.2. Active Compounds Found in Tri Garn Pis (TGP) Recipe Extract

**Figure 3:**
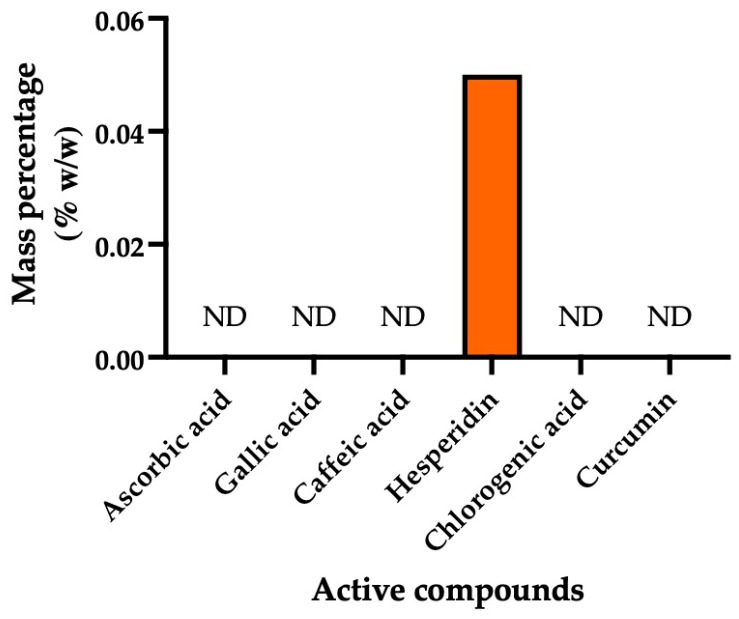
HPLC quantification of active compounds in TGP extract. Bar graph showing mass percentage (% *w*/*w*) of six bioactive compounds consisting of ascorbic acid, gallic acid, caffeic acid, hesperidin, chlorogenic acid, and curcumin. ND; not detected.

The active compounds in the TGP recipe extract, including ascorbic acid, gallic acid, caffeic acid, hesperidin, chlorogenic acid, and curcumin, were analyzed (Figure 3). The hesperidin was detected at 0.05 ± 0.00% *w*/*w*, whereas ascorbic acid, gallic acid, caffeic acid, chlorogenic acid, and curcumin were not detected (Figure 3).

### 3.3. The Effect of TGP Recipe Extract on Improving Depression-like Behaviors in DexCS-Treated Mice

**Figure 4:**
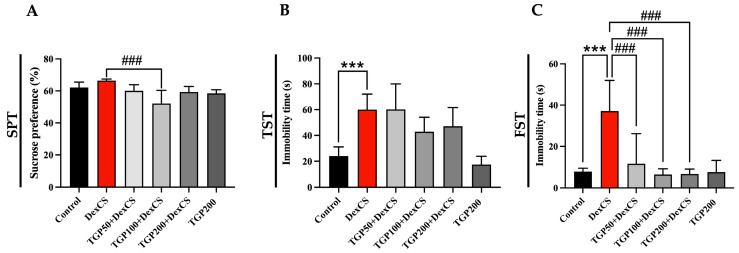
The sucrose preference test ((**A**); SPT) and immobility times of tail suspension test ((**B**); TST) and the forced swimming test ((**C**); FST), determined by depression-like behavior tests compared with control and DexCS groups. Significant difference (*** *p* < 0.001) compared with the control group. Significant difference (^###^ *p* < 0.001) compared with the DexCS group.

The results in Figure 4 confirm that the mice induced with dexamethasone (DEX) exhibited a chronic stress condition with depressive-like behavior, as revealed by significant increases in immobility. Significantly, the TGP recipe extract in co-treatment mice reduced the immobility time in the forced swimming test (FST) compared with the DexCS group (Figure 4C). In the sucrose preference test (SPT) for assessing anhedonia behavior, the TGP recipe extract at a dose of 100 mg/kg BW + DexCS decreased the percentage of sucrose preference, but no differences were observed in the other groups compared to the DexCS group (Figure 4A). Moreover, the immobility time in the tail suspension test in co-treatment mice showed no significant difference as compared with the DexCS group (Figure 4B).

### 3.4. Effect of TGP Recipe Extract on Enhanced Male Sexual Behaviors in DexCS-Treated Mice

**Figure 5:**
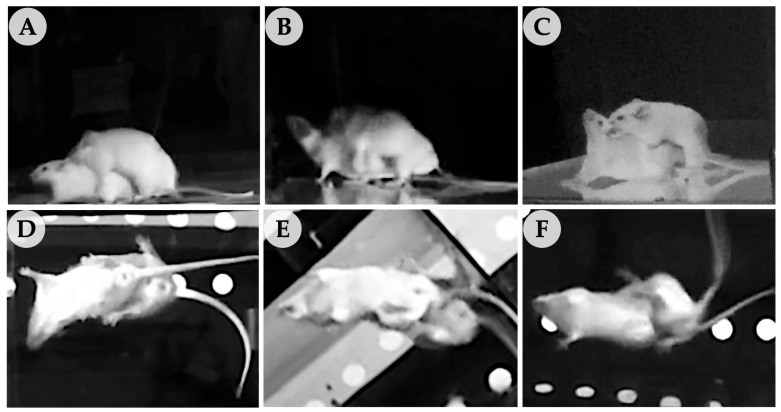
Sexual behavior of male mice observed using video recordings from the lateral (**A**–**C**) and ventral (**D**–**F**) views. Mounting (**A**,**D**); the back of the female placed by his front paws. Intromission (**B**,**E**): insertion of penis into the female’s vagina. Ejaculation (**C**,**F**): increased rate of thrusting after intromission, followed by self-cleaning.

**Figure 6:**
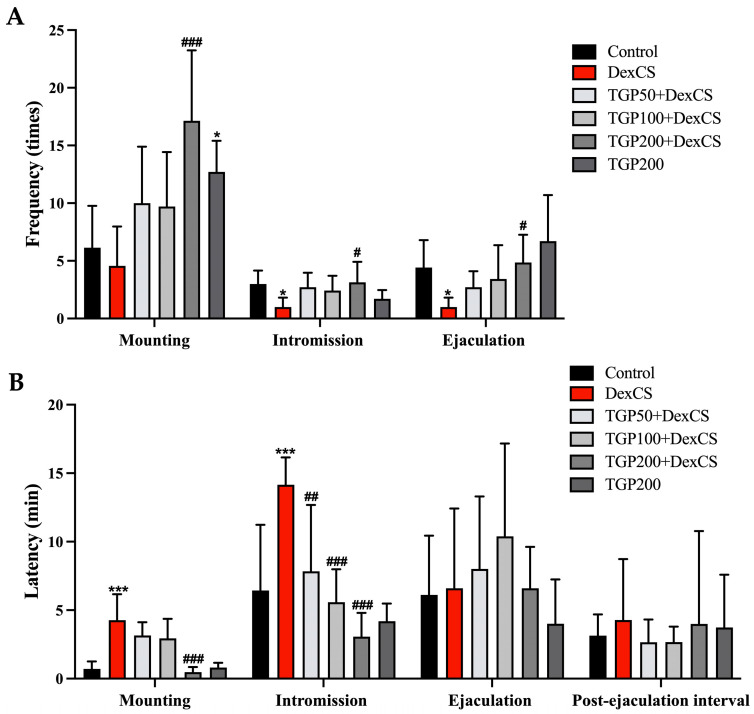
Frequency (**A**) and latency (**B**) of mounting, intromission, ejaculation, and post ejaculation interval behaviors compared among control, DexCS, and treated groups within 30 min after coupling with estrous female after 42 consecutive days of chronic stress induction. Significant difference (* *p* < 0.05, *** *p* < 0.001) compared with the control group. Significant difference (^#^ *p* < 0.05, ^##^ *p* < 0.01, ^###^ *p* < 0.001) compared with the DexCS group.

The sexual behaviors of male mice (mounting, intromission, and ejaculation) were observed using VDO recordings from lateral (Figure 5A–C) and ventral (Figure 5D–F) views. Following 42 days of DEX-induced chronic stress and TGP extract treatment, DexCS impaired sexual behavior, evidenced by a significant decrease in mount frequency (MF), intromission frequency (IF), and ejaculation frequency (EF), together with increases in mount latency (ML) and intromission latency (IL) compared to the control group (Figure 6A,B). Notably, the TGP recipe extract at 200 mg/kg BW + DexCS significantly increased MF, IF, and EF while decreasing ML and IL compared to the DexCS group (Figure 6A,B). In addition, IL was significantly decreased in mice treated with the TGP recipe extract at 50 and 100 mg/kg BW compared to the DexCS group (Figure 6B). Although the ejaculation latency (EL) and post-ejaculation interval (PEI) were not significantly different among groups, other sexual behaviors were improved after TGP treatment (Figure 6B).

### 3.5. Effect of TGP Recipe Extract on the Morphology and Weights of Body and Reproductive Organs in DexCS-Treated Mice

**Figure 7:**
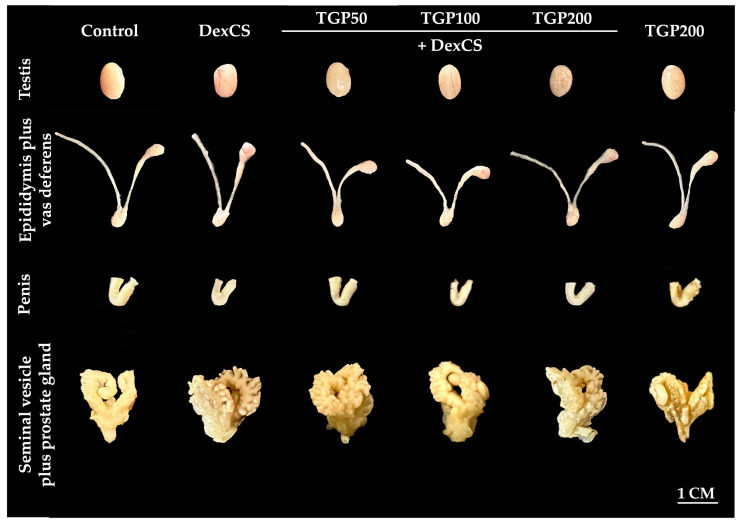
The morphological photographs of testis, epididymis plus vas deferens, penis, and seminal vesicle plus prostate gland of control, DexCS, TGPs (50, 100, and 200) + DexCS, and TGP200 groups.

The morphology of the reproductive organs, including the testis, epididymis plus vas deferens, penis, and seminal vesicle plus prostate gland, was not obviously changed in all experimental groups, as shown in Figure 7.

The results revealed that the weights of the body, testis, epididymis plus vas deferens, penis, and seminal vesicle plus prostate gland showed no significant differences among the control, DexCS, and treated groups (Table 2).

### 3.6. Effect of TGP Recipe Extract on Histology of Mouse Testis, Caudal Epididymis, Penis, and Seminal Vesicle in DexCS-Treated Mice

**Figure 8:**
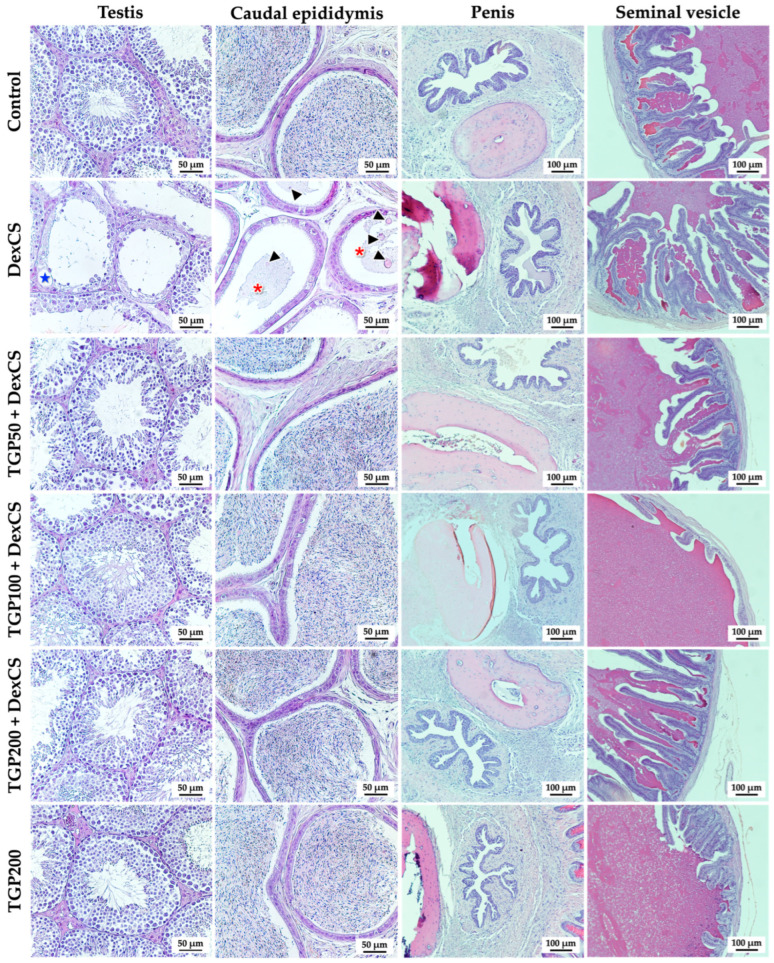
Histological photographs of the testis, epididymis, penis, and seminal vesicle of control, DexCS, TGPs (50, 100, and 200) + DexCS, and TGP200 groups stained with H&E. Blue star shows vacuolization, asterisks indicate the reduction in sperm mass within the caudal epididymal lumen, and arrow heads present the round cells.

The histology of the mouse testis, caudal epididymis, penis, and seminal vesicle in control, DexCS, TGPs (50, 100, and 200) + DexCS, and TGP200 groups is shown in Figure 8. Compared to the control, the testicular damages, including germ cell disorganization and vacuolization in seminiferous epithelium, were found in the DexCS group. In addition, the sperm mass within the lumens of some caudal epididymis was decreased, and the round cells were found in the DexCS group compared to the control group (Figure 8). Results show that the TGP recipe extract could ameliorate testicular and caudal epididymal damage in DexCS animals. No histopathological alterations were observed in the penis, and seminal vesicles were observed in co-treatment groups when compared to the control and DexCS mice (Figure 8).

**Figure 9:**
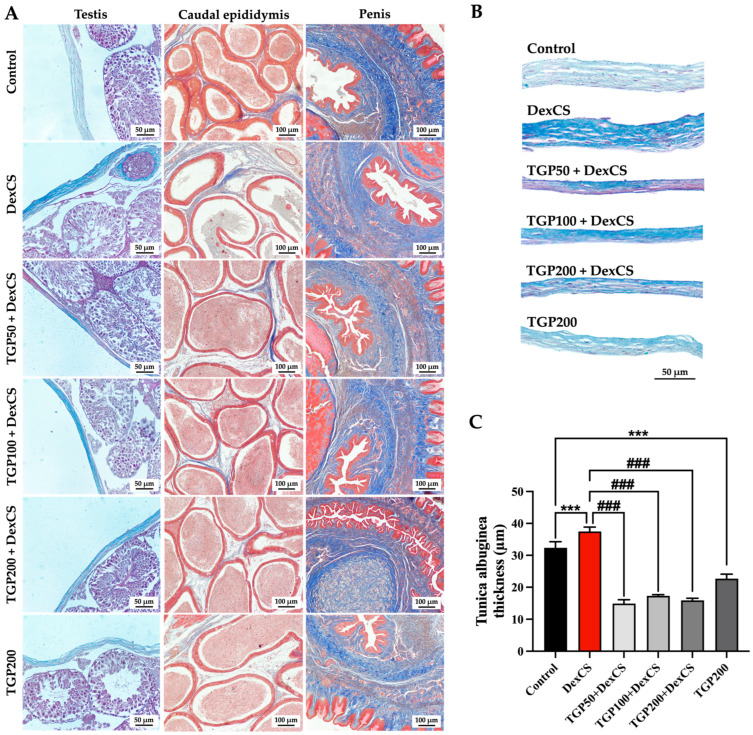
Histological photographs (**A**) of the testis, epididymis, penis, and seminal vesicle and tunica albuginea stained by Masson’s trichome (**B**) and quantitative analysis of its thickness compared among groups (**C**). Significant difference (*** *p* < 0.001) compared with the control group. Significant difference (^###^ *p* < 0.001) compared with the DexCS group.

The caudal epididymis, penis, and testis stained with Masson’s trichrome were observed under a light microscope and revealed that the tunica albuginea thickness was significantly increased in the DexCS group compared with the control group and significantly decreased in the co-treatment group compared with the DexCS group (Figure 9A–C). In addition, there were no significant differences in collagen fiber accumulation in the caudal epididymis and penis among the control, DexCS, all doses of TGPs (50, 100, and 200) + DexCS, and TGP200 groups, as shown in Figure 9A.

### 3.7. TGP Recipe Extract Improved Sperm Quality and Diameter and Epithelial Height of Seminiferous Tubule in DexCS-Treated Mice

**Figure 10:**
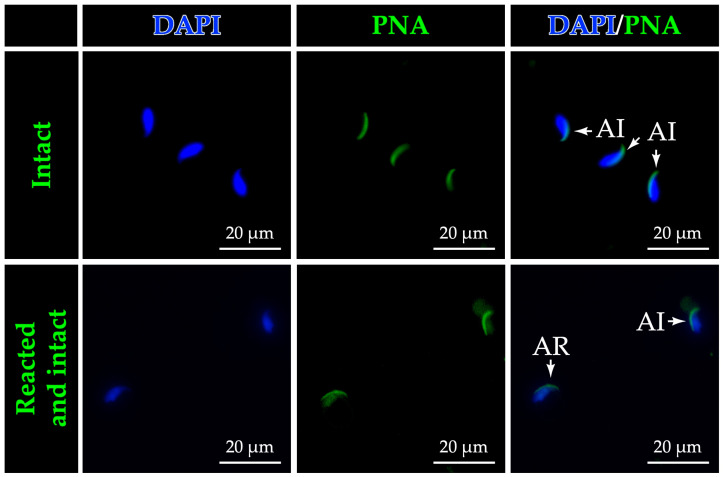
Photomicrographs showing the acrosomal status of mouse sperm cells labeled with PNA lectin, classified as having either intact (AI) or reacted (AR) acrosomes. DAPI was used as a blue fluorescent counterstain to visualize the nuclei, while positive immunoreactivity for PNA lectin appears as green fluorescence.

As shown in Table 3 and Figure 10, DexCS administration significantly decreased total sperm concentration (*p* < 0.05) and sperm viability (*p* < 0.001), while significantly increasing the percentage of acrosome-reacted sperm (labeled with PNA lectin), sperm abnormality, including total bent tail and total proximal droplet of sperm, compared to the control group. In addition, DexCS also significantly reduced the diameter and epithelial height of the seminiferous tubule (Table 3). Treatment with TGP could improve all epididymal damages caused by DexCS, as shown in Table 3.

**Figure 11:**
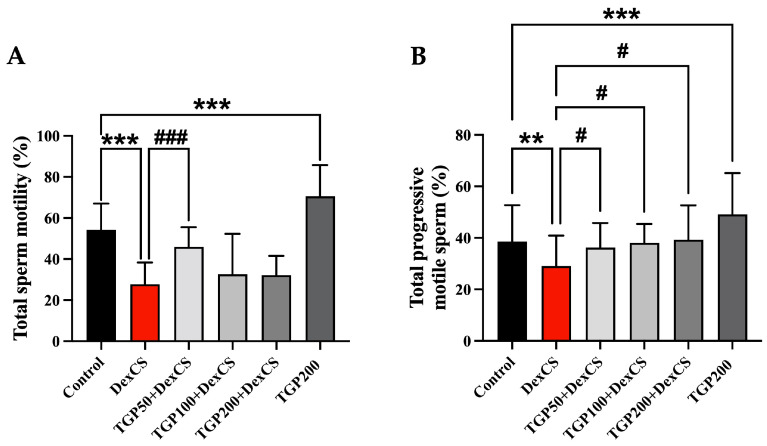
Photographs showing percentage of total sperm motility (**A**) and total progressive motile sperm (**B**) analyzed by CASA compared among control, DexCS, and TGPs (50, 100, and 200) + DexCS, and TGP200 groups. Significant difference (** *p* < 0.01, *** *p* < 0.001) compared with the control group. Significant difference (^#^ *p* < 0.05, ^###^ *p* < 0.001) compared with the DexCS group.

Sperm motility was assessed using CASA. DexCS-induced mice revealed a significant reduction in both percentage of total sperm motility and progressive motility (Figure 11A,B) compared to the control group. Treatment with TGP extract at 50 mg improved the percentage of total sperm motility, and all doses of TGP extract significantly increased the percentage of progressive motile sperm in DexCS-induced mice (Figure 11B). A high dose of TGP extract (200 mg) has no toxic effect on the sperm motility (Figure 11).

### 3.8. Effect of TGP Recipe Extract on the Testicular TyrPho Protein Expressions in DexCS-Treated Mice

**Figure 12:**
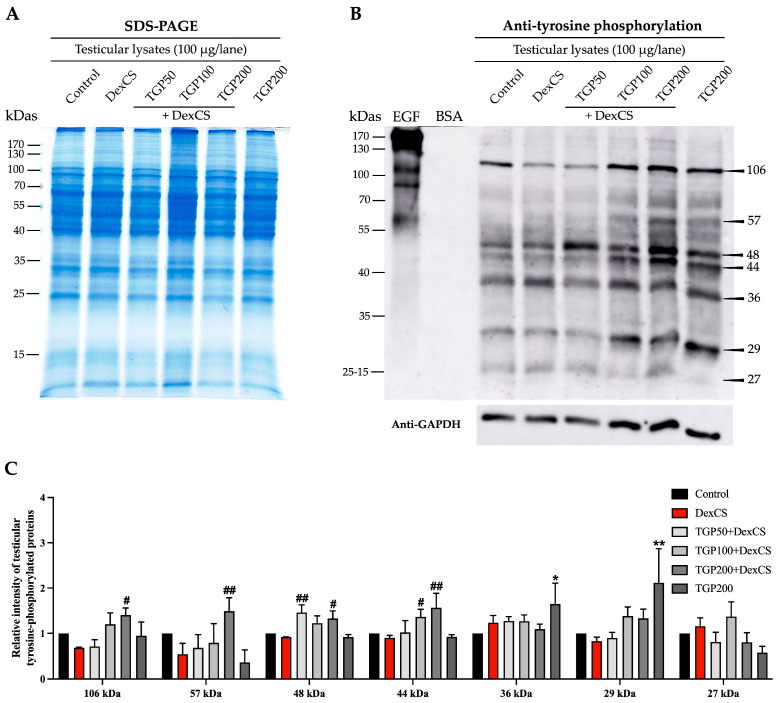
Profiles of total testicular proteins analyzed by SDS-PAGE (**A**), the expressions of testicular TyrPho proteins (**B**), and relative intensity of testicular TyrPho proteins (**C**) in comparison among control, DexCS, and treated groups. Epidermal growth factor (EGF)-like factor and bovine serum albumin (BSA) were used as positive and negative controls, respectively. Glyceraldehyde-3-phosphate dehydrogenase (GAPDH) was used as an internal control. kDa; kilodalton. Significant difference (* *p* < 0.05, ** *p* < 0.01) compared with the control group, Significant difference (^#^ *p* < 0.05, ^##^ *p* < 0.01) compared with the DexCS group.

Equal amounts of testicular proteins in all groups were confirmed using SDS-PAGE and GAPDH expression as the internal control (Figure 12A,B). The expressions of testicular TyrPho proteins were demonstrated. Although the relative intensity in the DexCS group was not different from that in the control group, treatment with TGP extract resulted in increased intensities of seven TyrPho bands (106, 57, 48, 44, 36, 29, and 27 kDa) as shown in Figure 12B. The results showed that TGP50 and 200 recipe extracts significantly increased the relative intensities of TyrPho proteins (106, 57, 48, and 44 kDa) in the testicular DexCS group (Figure 12C). Compared to the DexCS group, the expression of 44 kDa TyrPho protein was significantly increased in the TGP 100 mg/kg BW + DexCS group. Noticeably, the TyrPho expressions at 36 and 29 kDas were significantly increased in TGP200 alone mice compared to the control (Figure 12C). However, the relative intensities of the 27 kDa testicular TyrPho protein showed no significant differences in all groups compared to those of the control and DexCS groups (Figure 12C).

### 3.9. Effect of TGP Recipe Extract on Testicular Protein Expressions in DexCS-Treated Mice

**Figure 13:**
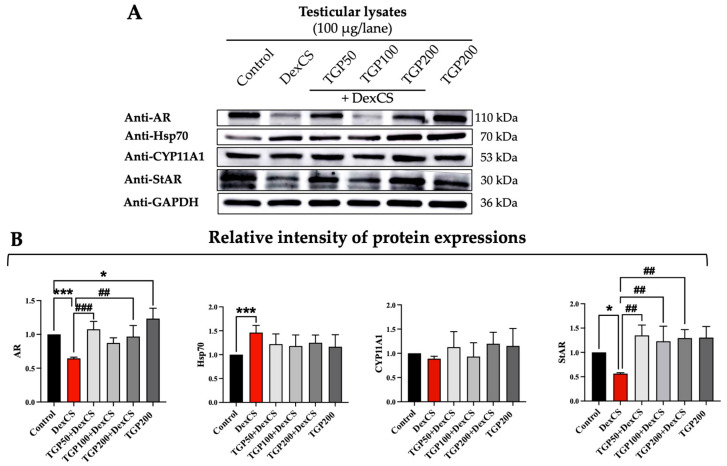
Testicular expressions of androgen receptor (AR), heat shock protein 70 (Hsp70), cytochrome P450 family 11 subfamily A member 1 (CYP11A1), and steroidogenic acute regulatory (StAR) proteins (**A**) and their relative intensities (**B**) in the control, DexCS, TGPs (50, 100, and 200) + DexCS, and TGP200 groups. Glyceraldehyde-3-phosphate dehydrogenase (GAPDH) used as an internal control. Significant difference (* *p* < 0.05, *** *p* < 0.001) compared with the control group. Significant difference (^##^ *p* < 0.01, ^###^ *p* < 0.001) compared with the DexCS group.

The results show that the androgen receptor (AR) and steroidogenic acute regulatory (StAR) proteins were significantly decreased in the testis of DexCS-treated mice. In contrast, the heat shock protein 70 (Hsp70) expression following testicular DexCS induction was significantly increased when compared to the control group (Figure 13A,B).

Treatment with TGP extracts dose-dependently (50, 100, and 200 mg/kg BW) improved the decreased expressions of AR and StAR proteins in DexCS-induced mice (Figure 13A,B). However, the expressions of heat shock protein 70 (Hsp70) and cytochrome P450 family 11 subfamily A member 1 (CYP11A1) were not obviously changed in all different doses of TGP + DexCS groups compared to DexCS or control group (Figure 13A,B).

### 3.10. Effect of TGP Recipe Extract on Testicular Apoptotic Protein Expressions in DexCS-Treated Mice

**Figure 14:**
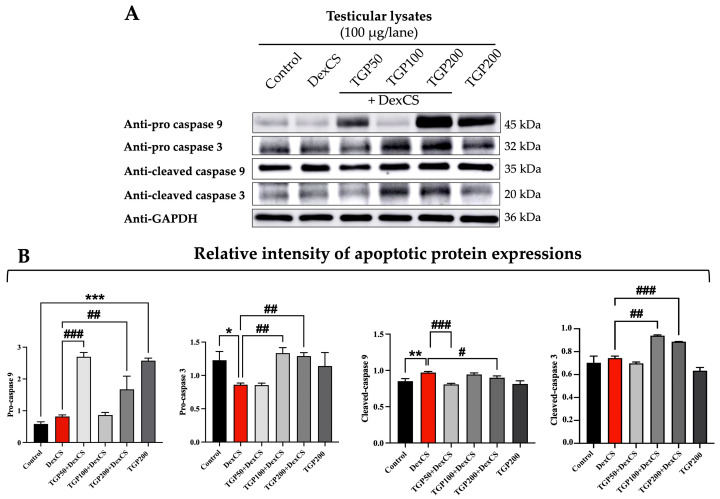
Apoptotic protein expressions (caspase-3 and -9 proteins) in testicular lysate (**A**) and their relative intensities (**B**) in the control, DexCS, TGPs (50, 100, and 200) + DexCS, and TGP200 groups. Glyceraldehyde-3-phosphate dehydrogenase (GAPDH) used as an internal control. Significant difference (* *p* < 0.05, ** *p* < 0.01, *** *p* < 0.001) compared with the control group. Significant difference (^#^ *p* < 0.05, ^##^ *p* < 0.01, ^###^ *p* < 0.001) compared with the DexCS group.

DexCS administration significantly decreased pro-caspase-3 expression in the testis and increased cleaved-caspase-9 expression compared to the control testis (Figure 14A,B). In contrast, the expressions of pro-caspase-9 and cleaved-caspase-3 did not differ significantly between the DexCS and control groups (Figure 14A,B). Results show that TGP 50 and 200 significantly increased pro-caspase-9 expression, while TGP 100 and 200 significantly increased pro-caspase-3 expression in DexCS testis. (Figure 14A,B). In contrast, the expression of cleaved-caspase-9 was significantly decreased in TGP 50 and 200 co-treatment groups, while cleaved-caspase-3 expression was significantly increased in TGP 100 and 200 co-treatment groups (Figure 14A,B).

## 4. Discussion

This study investigates the protective effects and underlying mechanisms of the Tri Garn Pis (TGP) extract in improving sexual performance and alleviating testicular dysfunction-related apoptosis induced by DexCS in mice. The results demonstrate that the TGP recipe ethanolic extract exhibits substantial antioxidant potential, as evidenced by its total phenolic and flavonoid contents, together with DPPH radical scavenging and FRAP activities. Similar findings have been reported from leaves and flowers of *Ocimum tenuiflorum* L. (Lamiaceae), rhizome extracts of *Curcuma longa* L., and *Boesenbergia rotunda* (Zingiberaceae), which contain the phytochemicals, particularly polyphenols and flavonoids, that act as potent antioxidants capable of modulating redox balance in biological systems [56,57,58,59,60].

Previous studies have demonstrated that DEX induces a depression-like phenotype, confirmed by a significant increase in immobility time in the tail suspension test (TST) and forced swimming test (FST) in animal models, consistent with our study indicating behavioral despair [11,12,61,62]. Our results revealed that TGP extract with antioxidants ameliorates depression-like behavior in mice induced by DexCS. Antioxidant activity derived from species in the same family as TGP may attenuate excess ROS and cortisol levels, leading to reduced neuroinflammation within the limbic system, especially in the hippocampus, and consequently reducing depression-like behavior [63,64].

This study is the first to investigate the protective effects of TGP extract on sexual behavior in DexCS mice. Chronic unpredictable mild stress (CUMS) and CS are associated with HPA axis hyperactivity and HPG axis suppression, characterized by elevated corticosterone and reduced testosterone levels, which diminish sexual desire and motivation due to stress-induced neuroendocrine alterations [4,65]. In addition, testosterone may subsequently influence neuronal activity via neurotransmitter release in the brain areas such as gamma-aminobutyric acid (GABA) in the medial pre-optic area (MPOA) in the hypothalamus, as dopamine, norepinephrine, and acetylcholine, all of which are involved in regulating copulatory behavior [32,36,37,38,39]. We found that TGP extract ameliorated DexCS-induced impairments in male sexual behavior in DexCS mice. Consistent with previous studies, the extract from the rhizomes of *Kaempferia parviflora* (Zingiberaceae) improved testosterone level and sexual performance parameters (ML, MF, EL, and PEI), and erectile dysfunction [66,67,68]. Previous studies have reported that quercetin and aglycone, a flavonoid and antioxidant found in individual plants, can improve Leydig cell damage, resulting in enhancing testosterone synthesis [32,36,37]. Interestingly, a high dose of TGP restored Leydig cell function, as evidenced by the increased testicular StAR and androgen receptor (AR) protein expressions, which are assumed to stimulate testosterone production, enhance sexual performance (Figure 6A,B), and increase aphrodisiac potential in DexCS mice. Because serum volumes were insufficient, we were unable to measure circulating testosterone, LH, or FSH. This limits our ability to fully assess endocrine mediation of the observed testicular and sperm outcomes and should be addressed in future studies. Moreover, lifestyle factors including nutrition, smoking, obesity and physical activity can influence male reproductive health. These factors have been associated with altered semen parameters in humans [69,70,71,72]. Although physical activity was not a variable in our animal model, the multifactorial nature of male infertility implies that any translational application of TGP should consider coexisting lifestyle influences and that clinical studies should control for exercise and other behaviors.

Recent studies have demonstrated that DexCS induces testicular histopathological alterations, characterized by damage of the seminiferous epithelium, including germ cells, Sertoli cells, and Leydig cells, resulting in reduced testosterone levels and increased testicular apoptosis [12,68]. Consistent with our report, DexCS reduced both the diameter and epithelial height of the seminiferous tubule, indicating testicular morphological damage and impaired spermatogenesis. The histological examination in this study revealed that TGP extract improves testicular and epididymal histopathology induced by DexCS, evidenced by reduced germ cell disorganization and vacuolization, and restoration of sperm mass within the caudal epididymis (Figure 8). TGP may exert a protective effect on testicular damage following glucocorticoid-induced damage, consistent with a previous study [36]. This protective effect is likely mediated through antioxidant defense mechanisms, reducing oxidative stress and promoting the recovery of the germinal epithelium. Excess ROS caused by DEX drives testicular fibrosis through activation of the TGF-β/Smad signaling pathway, increasing collagen fiber synthesis and deposition, a process implicated in male reproductive dysfunction [73,74]. TGP treatment at all doses could normalize the increased thickness of tunica albuginea by balancing ROS and antioxidant levels, leading to decreased collagen fiber accumulation in DexCS mice (Figure 9A–C).

DexCS and CS exposure promotes HPA axis activation and oxidative stress, which impairs synthesis of essential steroidogenic proteins such as androgen receptor (AR), steroidogenic acute regulatory protein (StAR), heat shock protein 70 (Hsp70), and cytochrome P450 family 11 subfamily A member 1 (CYP11A1) [4,5,6,7]. Recent studies have revealed that DexCS significantly decreased serum testosterone levels [68], resulting in reduced sperm quality parameters; notably, a reduction in progressive motility [12] and low sperm mass in the cauda epididymis [12,61]. In this study, TGP extract may stimulate steroidogenesis in Leydig cells and epithelial germ cell damage, which correlated with increased sperm quality, including total sperm concentration, progressive motility, viability, including reductions in reacted acrosome (labeled with PNA lectin), and abnormal sperm under DexCS (Figure 10). Consistent with previous studies, antioxidants in Wuzi Yanzong Pills (WZYZP) have been shown to restore testicular histopathological integrity associated with reduced germ cell apoptosis and improve poor sperm quality under testicular heat stress [75,76]. Notably, improvements in total sperm count and motility were most evident at the lowest TGP dose (50 mg/kgBW), whereas 100 and 200 mg/kgBW did not produce comparable effects. This non-monotonic pattern is consistent with a biphasic response reported for several phytochemicals [77,78]. Possible explanations include dose-dependent shifts in redox balance, transport pathways, or receptor desensitization at higher doses. These processes may differentially affect sperm maturation and motility. Further dose–response and mechanistic studies are needed to clarify these effects. In addition, environmental pollutants exert reproductive toxicity via induction of oxidative stress, increasing lipid peroxidation and compromising sperm DNA and membrane integrity [22]. Polyphenolic constituents of herbal extracts can directly scavenge free radicals, chelate transition metals, modulate redox-sensitive signaling pathways, and upregulate endogenous SOD, catalase, and GPx [79,80,81,82]. Although we did not measure these markers here, the observed reductions in caspase activation and preservation of acrosomal integrity are consistent with reduced oxidative injury following TGP exposure. Therefore, TGP may mitigate oxidative damage, inferred from improved acrosomal integrity and reduced apoptosis.

CS and chemical challenge lead to changes in the pattern and expression of testicular TyrPho proteins and decrease epididymal sperm lectin in animals, affecting the structural and functional sperm related to premature capacitation and acrosome reaction, causing low fertilizing capacity [7,65,83]. In this study, DexCS tended to decrease TyrPho proteins in the testis, suggesting that it impacts spermatogenesis and spermiogenesis, leading to disturbed structural sperm maturation, which is related to increased sperm abnormality, leading to low fertilization efficiency. Herbal medicinal plants have been shown to normalize testicular TyrPho levels in CS-induced rats [26,84,85]. In addition, polyherbal formulations rich in antioxidants ameliorate the imbalance in testicular homeostasis via suppressed cGAS-STING signaling pathway, resulting in testicular inflammation and sperm development [75]. Interestingly, all doses of TGPs from our study increased testicular TyrPho protein expressions (106, 57, 48, and 44 kDa) in DexCS mice, indicating that TGPs may improve testicular damage and poor sperm quality, especially progressive motility and acrosome-reacted sperm (PNA lectin binding), by modulating phosphorylation-dependent regulatory pathways.

DEX-induced testicular oxidative stress reduces gene expressions of CYP11A1 and StAR, both key components for testosterone biosynthesis [86].

In this study, AR and StAR protein intensities, both suppressed by DexCS, were upregulated by TGP, while heat shock protein 70 (Hsp70) and CYP11A1 levels remained unchanged, suggesting a direct effect of DexCS on StAR and AR that leads to disruption of steroidogenesis (Figure 13A,B). Similarly to previous research, red ginseng supplementation enhances the mRNA expression levels of CYP11A1 and StAR in testicular tissue after DEX-induced oxidative stress [86]. Our results demonstrate that antioxidant properties from TGPs may enhance steroidogenesis in Leydig cells by upregulating testicular StAR expression, facilitating the conversion of cholesterol into testosterone, thereby leading to increased testosterone levels and improved sperm quality under DexCS exposure.

It has been reported that DexCS significantly increased testicular cleaved caspase-3 and caspase-9 expressions, thereby promoting testicular apoptosis in male mice [12]; our findings are consistent with this report (Figure 14A,B). DexCS decreased pro-caspase-3 and increased cleaved caspase-9 expression, indicating enhanced apoptosis in the testis (Figure 14A,B). TGP at various doses could increase pro-caspase-3 and -9 while decreasing cleaved caspase-9 (Figure 14A,B). Consistent with previous studies, the revealed regulation of caspase expression suggests that bioactive compounds from single plant extracts may modulate the intrinsic apoptotic pathway in the CUMS model by suppressing cleaved caspase-3 and -9 while promoting pro-caspase forms [4,87]. TGP may attenuate apoptosis by inhibiting caspase activation and preserving the precursor (pro-) forms of caspase-3 and caspase-9, similar to inhibitors that block apoptosis by preventing the cleavage of pro-caspases into their active forms, thereby improving testicular degeneration and protecting poor sperm quality by reducing excessive germ cell apoptosis typically associated with caspase activation.

## 5. Conclusions

TGP extract appears to counteract DexCS-induced oxidative stress and reproductive dysfunction through various mechanisms, including enhancement of antioxidant capacity, modulation of behavioral and sexual functions, restoration of sperm quality, improvement of testicular structure, and regulation of testicular protein expressions related to androgen steroidogenesis and apoptosis. These findings highlight TGP as a promising alternative for further investigation in the context of male reproductive health, particularly for the treatment of conditions such as stress-induced testicular pathology and male infertility.

## Figures and Tables

**Table 1:** Summary of potential antioxidants and antioxidant capacity of Tri Garn Pis (TGP) recipe extract.

	Potential Antioxidants		Antioxidant Capacities	
	Total Phenolic Content (mg GAE/g TGP)	Flavonoid Content (mg Catechin/g TGP)	DPPH: IC_50_ (mg/mL)	FRAP Value (μmol of Fe (II)/g TGP)
TGP recipe extract	15.86 ± 0.27	6.68 ± 0.17	644.87 ± 2.73	151.25 ± 0.13

TGP; Tri Garn Pis recipe extract, GAE; gallic acid equivalent, DPPH; 2,2-diphenyl-1-picrylhydrazyl, IC_50_; half maximal inhibitory concentration, and FRAP; ferric reducing antioxidant power.

**Table 2:** Comparison of body and reproductive organ weights among control, DexCS, and treated mice after 42 consecutive days of chronic stress induction.

Parameters	Groups					
	Control	DexCS	TGP50	TGP100	TGP200	TGP200
			DexCS			
Pre-DexCS BW (g)	43.16 ± 3.62	43.14 ± 3.12	43.13 ± 3.63	43.18 ± 4.62	43.15 ± 4.23	43.13 ± 3.13
Post-DexCS BW (g)	47.12 ± 4.96	47.88 ± 4.64	47.88 ± 4.64	45.16 ± 5.60	46.38 ± 6.21	46.31 ± 3.56
Changed body weight (%)	9.10 ± 1.58	10.87 ± 1.54	11.39 ± 3.77	5.85 ± 0.45	7.26 ± 2.11	7.44 ± 1.38
Testis						
Absolute weight (g)	0.133 ± 0.015	0.136 ± 0.016	0.133 ± 0.013	0.130 ± 0.015	0.133 ± 0.016	0.140 ± 0.012
Relative weight (g/100 g)	0.302 ± 0.036	0.302 ± 0.041	0.307 ± 0.039	0.308 ± 0.051	0.306 ± 0.048	0.320 ± 0.029
Epididymis plus vas deferens						
Absolute weight (g)	0.068 ± 0.006	0.069 ± 0.004	0.070 ± 0.006	0.066 ± 0.006	0.069 ± 0.006	0.068 ± 0.007
Relative weight (g/100 g)	0.154 ± 0.015	0.154 ± 0.016	0.161 ± 0.016	0.156 ± 0.024	0.159 ± 0.024	0.156 ± 0.020
Penis						
Absolute weight (g)	0.070 ± 0.007	0.063 ± 0.007	0.068 ± 0.009	0.060 ± 0.006	0.063 ± 0.006	0.066 ± 0.005
Relative weight (g/100 g)	0.161 ± 0.026	0.140 ± 0.016	0.157 ± 0.021	0.144 ± 0.022	0.146 ± 0.026	0.155 ± 0.021
Seminal vesicle plus prostate gland						
Absolute weight (g)	0.530 ± 0.110	0.644 ± 0.130	0.668 ± 0.099	0.597± 0.139	0.590 ± 0.131	0.531 ± 0.091
Relative weight (g/100 g)	1.204 ± 0.224	1.429 ± 0.313	1.533 ± 0.223	1.381 ± 0.218	1.254 ± 0.300	1.212 ± 0.173

Data were represented as mean ± standard deviation (S.D.), TGP; Tri Garn Pis recipe extract, DexCS; dexamethasone-induced chronic stress, BW; body weight, g; gram.

**Table 3:** Comparisons of the seminiferous tubule (diameter and epithelium height) and sperm parameters among control, DexCS, and treated mice after 42 consecutive days of chronic stress induction.

Parameters	Groups					
	Control	DexCS	TGP50	TGP100	TGP200	TGP200
			DexCS			
Total sperm concentration (10^6^/mL)	17.29 ± 4.47	13.28 ± 6.50 *	16.71 ± 5.28 ^#^	10.10 ± 2.61	12.38 ± 3.61	27.10 ± 9.46 ***
Sperm parameters						
Sperm viability (%)	96.90 ± 1.39	81.90 ± 2.22 ***	94.50 ± 1.96 ^###^	96.63 ± 1.03 ^###^	96.67 ± 0.29 ^###^	97.83 ± 0.29
Acrosome reaction (%)	2.00 ± 0.00	13.50 ± 2.12 ***	4.60 ± 0.82 ^###^	4.20 ± 0.97 ^###^	3.80 ± 0.57 ^###^	3.90 ± 1.14
Sperm abnormalities						
Total bent tail of sperm (%)	5.26 ± 2.48	6.85 ± 3.16 *	4.95 ± 2.26 ^##^	3.73 ± 1.84 ^###^	3.31 ± 1.15 ^###^	5.28 ± 2.91
Total proximal droplet of sperm (%)	16.49 ± 2.00	28.34 ± 9.80 **	28.10 ± 7.54	27.83 ± 6.72	25.32 ± 8.19	18.48 ± 7.21
Seminiferous tubule						
Diameter (μm)	230.61 ± 3.76	207.95 ± 2.98 ***	235.08 ± 2.67 ^###^	239.36 ± 2.75 ^###^	243.26 ± 2.35 ^###^	245.61 ± 2.53 **
Epithelium height (μm)	83.64 ± 2.77	50.18 ± 2.55 ***	74.91 ± 2.34 ^###^	79.63 ± 2.48 ^###^	79.36 ± 2.67 ^###^	82.83 ± 2.52

Data are represented as mean ± standard deviation (S.D.), TGP; Tri Garn Pis recipe extract, DexCS; dexamethasone-induced chronic stress, μm; micrometer. Significant difference (* *p* < 0.05, ** *p* < 0.01, *** *p* < 0.001) compared with the control group. Significant difference (^#^ *p* < 0.05, ^##^ *p* < 0.01, ^###^ *p* < 0.001) compared with the DexCS group.

## Data Availability

The data are not publicly accessible due to an ongoing study. However, the data used in this study can be obtained upon request from the corresponding author.

## Supplementary Materials

The following supporting information can be downloaded at https://www.mdpi.com/article/10.3390/life16010116/s1.
