# Supplementary material for: Preventive Effects of Tri Garn Pis Polyherbal Extract on Sexual Performance, Testicular Apoptosis, and Sperm Quality in a Dexamethasone-Induced Chronic Stress in Mice
Source: Life (Basel). 2026 Jan 13;16(1):116. doi: 10.3390/life16010116 (PMC12843107; doi:10.3390/life16010116)
Supplement: Supplementary file 1 [file life-16-00116-s001.zip › life-4068822 - supplementary - final/Supplementary File S1.pdf]

ANALYTICAL REPORT

Sample Name: TGP (Tri Garn Pis)

Service Type: Active compound quantification

Test Methods:
1. Total flavonoid content determination according to Wolfe et al. (2003)
2. Total phenolic content determination according to Wolfe et al. (2003)
3. Antioxidant activity assessment using DPPH assay according to Zhu et al. (2006)
4. Antioxidant activity assessment using FRAP assay according to Kubola and Siriamornpun (2008)

Report Date: March 30, 2023

Laboratory: Herbal Technology and Bioactive Compounds Laboratory
Institute of Research and Development of Agricultural and Agro-Industrial Products
Kasetsart University
50 Ngamwongwan Road, Lat Yao, Chatuchak, Bangkok 10900, Thailand
Tel: +66-2-942-8600-3 ext. 402 | Fax: +66-2-942-8600-3 ext. 407

RESULTS

1. Total Flavonoid Content

Table 1. Total flavonoid content

Sample	Flavonoid content (mg catechin/g sample)			
	Replicate 1	Replicate 2	Replicate 3	Average
TGP (Tri Garn Pis)	6.863	6.526	6.637	6.675

2. Total Phenolic Content

Table 2. Total phenolic content

Sample	Total phenolic (mg gallic acid/g sample)			
	Replicate 1	Replicate 2	Replicate 3	Average
TGP (Tri Garn Pis)	15.595	15.863	16.132	15.863

3. DPPH Radical Scavenging Activity

Table 3. DPPH radical scavenging capacity of TGP (Tri Garn Pis)

Sample	DPPH (IC_{50}, mg/ml)			
	Replicate 1	Replicate 2	Replicate 3	Average
TGP (Tri Garn Pis)	641.723	646.446	646.446	644.871
Ascorbic acid	4.426	4.470	4.492	4.463
Alpha tocopherol	18.390	17.732	18.102	18.075
BHT	121.274	121.937	124.051	122.420

4. Ferric Reducing Antioxidant Power (FRAP) Assay

Table 4. FRAP values of TGP (Tri Garn Pis)

Sample	FRAP value (μmol Fe(II)/1g sample)			
	Replicate 1	Replicate 2	Replicate 3	Average
TGP (Tri Garn Pis)	151.133	151.219	151.392	151.248
Ascorbic acid	12,460.661	12,519.454	12,692.374	12,557.496
Alpha tocopherol	3,290.680	3,344.285	3,389.244	3,341.403
BHT	2,251.427	2,344.804	2,415.701	2,337.311

APPENDIX
Determination of Flavonoid and Phenolic Contents and Antioxidant Activity
Chemicals and Equipment

1. Chemicals
- Catechin
- Sodium nitrite ($NaNO_2$)
- Sodium hydroxide (NaOH)
- Aluminum chloride ($AlCl_3$)
- Reverse osmosis (RO) water
- Gallic acid
- Sodium carbonate (Na_2CO_3)
- Folin-Ciocalteu reagent
- 2,2-Diphenyl-1-picrylhydrazyl (DPPH)
- 2,4,6-Tris(2-pyridyl)-s-triazine (TPTZ)
- Sodium acetate trihydrate ($CH_3COONa \cdot 3H_2O$)
- Acetic acid (CH_3COOH)
- Hydrochloric acid (HCl)
- Ferric chloride hexahydrate ($FeCl_3 \cdot 6H_2O$)
- Ferrous sulfate heptahydrate ($FeSO_4 \cdot 7H_2O$)
- Butylated hydroxytoluene (BHT)
- Ascorbic acid
- Alpha-Tocopherol

2. Equipment
- Test tubes
- Vortex mixer
- Autopipette / Automatic pipette
- Pipette tips
- Analytical balance
- Spectrophotometer

Methods

1. Determination of Total Flavonoid Content

Total flavonoid content was determined according to the method of Wolfe et al. (2003). Briefly, 250 µl of test sample was mixed with 1,250 µl of distilled water and 75 µl of 5% sodium nitrite solution. The mixture was allowed to stand for 5 min at room temperature. Subsequently, 150 µl of 10% aluminum chloride solution was added, and the mixture was incubated for 6 min. Then, 500 µl of 1 M sodium hydroxide and 275 µl of distilled water were added. The absorbance was measured at 510 nm using a spectrophotometer. All measurements were performed in triplicate. Total flavonoid content was calculated from the absorbance values using a catechin standard calibration curve and expressed as milligrams of catechin equivalents per gram of sample (mg catechin/g sample).

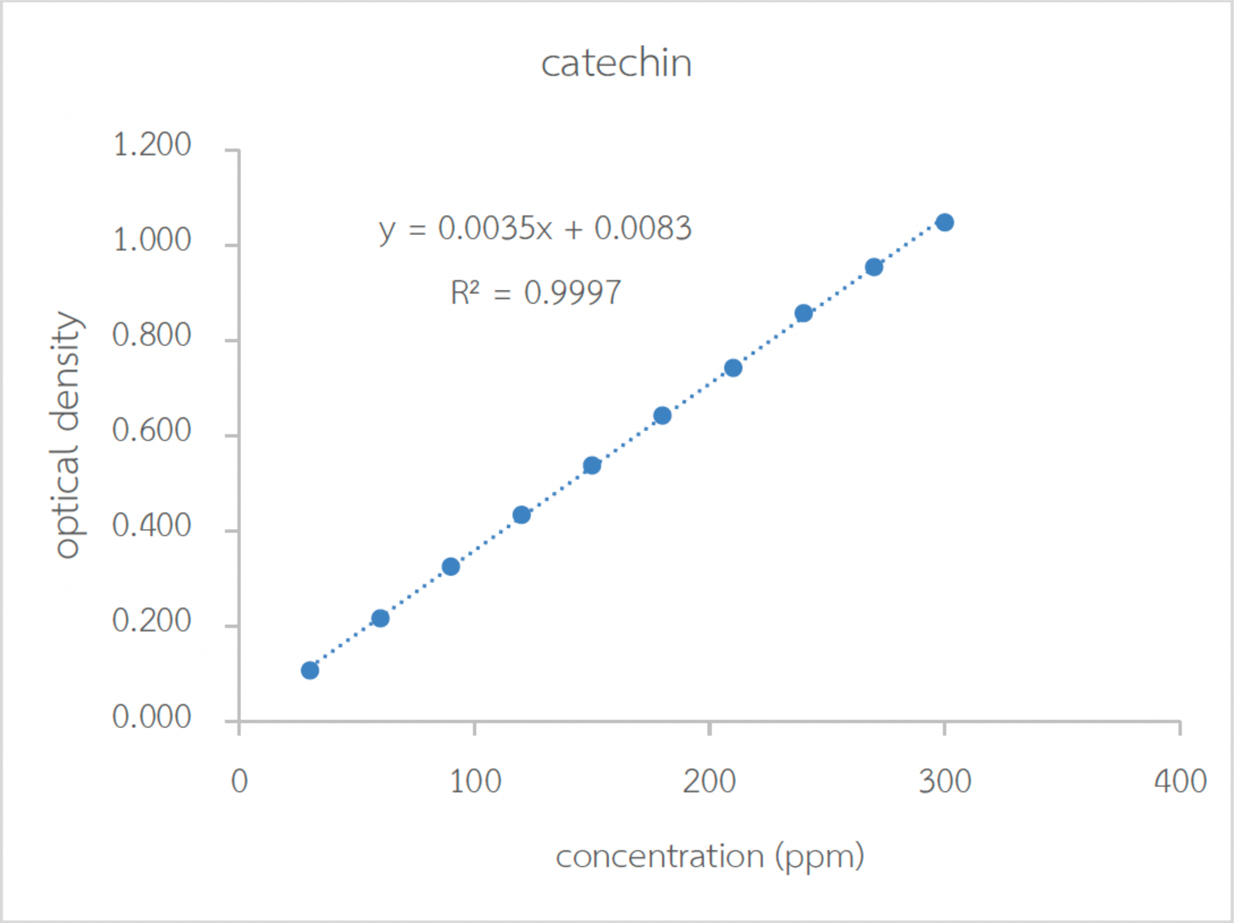

The catechin standard calibration curve was prepared at concentrations of 30, 60, 90, 120, 150, 180, 210, 240, 270, and 300 µg/ml.

2. Determination of Total Phenolic Content

Total phenolic content was analyzed using a modified Folin-Ciocalteu colorimetric method according to Wolfe et al. (2003). The extract was diluted to a concentration of 0.01 mg/ml. An aliquot of 125 µl of sample was pipetted into a test tube containing 500 µl of distilled water, followed by addition of 125 µl of Folin-Ciocalteu reagent. The mixture was allowed to stand for 6 min at room temperature. Subsequently, 1,250 µl of 7% sodium carbonate solution and 1,000 µl of distilled water were added. The mixture was incubated at room temperature for 90 min in the dark. Absorbance was measured at 760 nm using a spectrophotometer. Total phenolic content was calculated using a gallic acid standard calibration curve prepared at concentrations ranging from 30 to 270 µg/ml and expressed as milligrams of gallic acid equivalents per gram of sample (mg GAE/g sample).

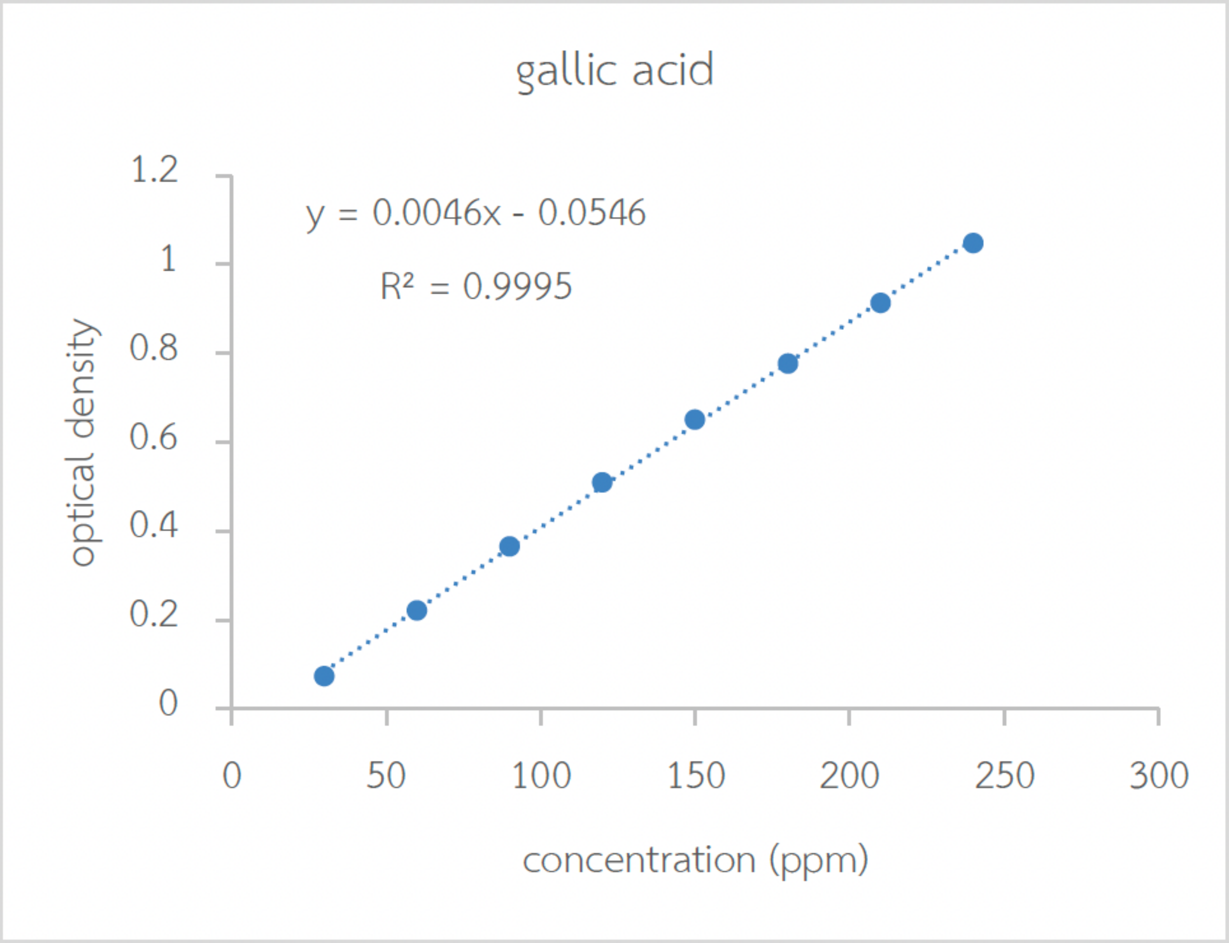

A gallic acid standard calibration curve was constructed at concentrations ranging from 30 to 240 µg/mL (30, 60, 90, 120, 150, 180, 210, and 240 µg/ml).

3. DPPH Radical Scavenging Activity Assay

The antioxidant activity was evaluated using the 2,2-diphenyl-1-picrylhydrazyl (DPPH) radical scavenging assay. Test samples were prepared at various concentrations. One milliliter of each sample concentration was mixed with 1 mL of 0.1 mM DPPH solution in 95% ethanol. The mixture was vortexed and incubated in the dark at room temperature for 30 min. Absorbance was measured at 517 nm using a spectrophotometer. All experiments were performed in triplicate. Ascorbic acid, α-tocopherol, and butylated hydroxytoluene (BHT) were used as positive controls. The DPPH radical scavenging activity was calculated using equation (1):

$$\text{DPPH radical scavenging activity (\%)} = [(A_0 - A_1)/A_0] \times 100 \quad (1)$$

where

A_0 is the absorbance of the control and

A_1 is the absorbance of the test sample.

The percentage of radical scavenging activity at different concentrations was plotted to determine the IC_{50} value, defined as the concentration of the test sample required to scavenge 50% of DPPH radicals.

4. Ferric Reducing Antioxidant Power (FRAP) Assay

The FRAP assay was performed according to the method of Kubola and Siriamornpun (2008). FRAP reagent was prepared by mixing 300 mM acetate buffer (pH 3.6), 20 mM $FeCl_3 \cdot 6H_2O$ solution, and 10 mM TPTZ (2,4,6-tris(2-pyridyl)-s-triazine) solution in 40 mM HCl at a ratio of 10:1:1 (v/v/v). The FRAP reagent was warmed to 37°C before use. Test sample solution was mixed with distilled water and FRAP reagent in a test tube. The mixture was incubated at 37°C for 4 min, and absorbance was measured at 593 nm using a spectrophotometer. All experiments were performed in triplicate. The antioxidant capacity was calculated from the absorbance values using a ferrous sulfate ($FeSO_4 \cdot 7H_2O$) standard calibration curve and expressed as micromoles of Fe(II) equivalents per gram of sample (µmol Fe(II)/1g sample).

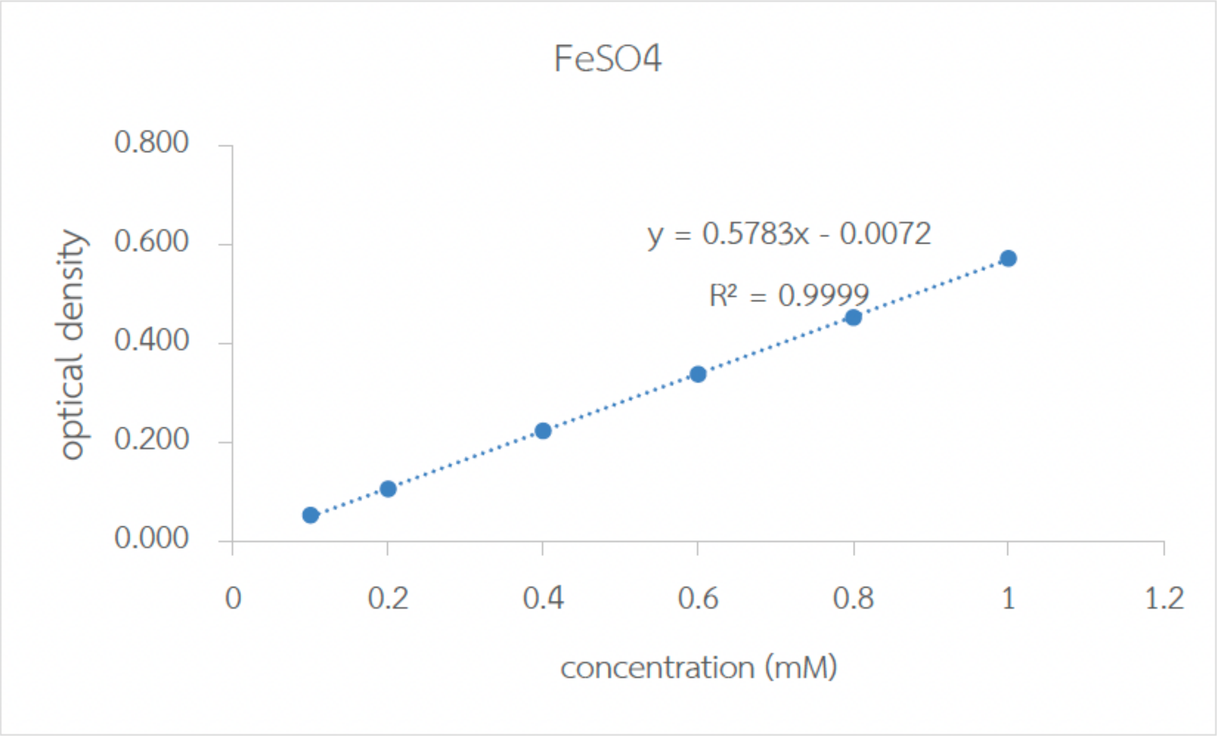

A ferrous sulfate standard calibration curve was constructed at concentrations ranging from 0.1 to 1.0 mM (0.1, 0.2, 0.4, 0.6, 0.8, and 1.0 mM).

References

Kubola, J. and Siriamornpun, S., 2008. Phenolic contents and antioxidant activities of bitter gourd (Momordica charantia L.) leaf, stem and fruit fraction extracts in vitro. *Food chemistry*, *110*(4), pp.881-890.

Wolfe, K., X. Wu and R. H. Liu. 2003. Antioxidant activity of apple peels. J. Agric. Food Chem. 51: 609-614.

Zhu, K., Zhou, H. and Qian, H., 2006. Antioxidant and free radical-scavenging activities of wheat germ protein hydrolysates (WGPH) prepared with alcalase. *Process Biochemistry*, *41*(6), pp.1296-1302.
