# Supplementary material for: Preventive Effects of Tri Garn Pis Polyherbal Extract on Sexual Performance, Testicular Apoptosis, and Sperm Quality in a Dexamethasone-Induced Chronic Stress in Mice
Source: Life (Basel). 2026 Jan 13;16(1):116. doi: 10.3390/life16010116 (PMC12843107; doi:10.3390/life16010116)
Supplement: Supplementary file 1 [file life-16-00116-s001.zip › life-4068822 - supplementary - final/Supplementary File S2.pdf]

FM-KAPI-TR

KASETSART UNIVERSITY KAPI (Kasetsart Agricultural and Agro-Industrial Product Improvement Institute) Office Address: Teera Sootabutra Building and Agro-Industrial Building 3, No. 50 Ngamwongwan Road, Ladyao, Chatuchak, Bangkok 10900 Tel: (+66)2 942 8600 ext. 207 Website: www.kapi.ku.ac.th E-mail: Kapi@ku.th

Test Report

**Report**	**Test report KAPI-TR FHC-NIR 2512-07**	**Date 091268**
Submission	Submission KAPI-TR FHC-NIR 2512-07	Date 091268

Name: Prof. Sitthichai Iamsa-ard, Department of Anatomy, Faculty of Medicine, Khon Kaen University**Address:** 123 Moo 16, Mittraphap Rd., Nai Muang Sub-district, Mueang Khon Kaen District, Khon Kaen Province 40002 **Tax ID:** 0994000391528

Summary Table of Results

**Test Item**	**Sample**	**Assays**
**Vitamin C Content (%w/w)**	ND	**High Performance Liquid Chromatography** (SHIMADZU; Nexera LC-40 series), Inert Sustain C18 column (4.6 mm ID x 250 mm, 5 µm), **Mobile phase:** (A) 0.1% Acetic acid, (B) Methanol at A:B ratio of 80:20, **Flow rate:** 0.6 ml/min, **Temp:** 30°C
**Gallic Acid Content (%w/w)**	ND	**High Performance Liquid Chromatography** (SHIMADZU; Nexera LC-40 series), Inert Sustain C18 column (4.6 mm ID x 250 mm, 5 µm), **Mobile phase:** (A) 1% Acetic acid, (B) Acetonitrile; utilizing a gradient elution system as follows: Mobile phase (A) at 0-28 min: 90%, 28-39 min: 60%, 39-50 min: 40%, 50-55 min: 10%, 55-70 min: 90%, **Flow rate:** 0.7 ml/min, **Temp:** 30°C
**Caffeic Acid Content (%w/w)**	ND	*Same test method as the item above*

Test Item	Sample	Assays
**Hesperidin Content (%w/w)**	**0.05 ± 0.00**	**High Performance Liquid Chromatography** (SHIMADZU; Nexera LC-40 series), Inert Sustain C18 column (4.6 mm ID x 250 mm, 5 µm), **Mobile phase:** (A) 0.1% Acetic acid, (B) Acetonitrile at A:B ratio of 80:20, **Flow rate:** 1.0 ml/min, **Temp:** 30°C
**Chlorogenic Acid Content (%w/w)**	ND	**High Performance Liquid Chromatography** (SHIMADZU; Nexera LC-40 series), Inert Sustain C18 column (4.6 mm ID x 250 mm, 5 µm), **Mobile phase:** (A) 1% Acetic acid, (B) Acetonitrile; utilizing a gradient elution system as follows: Mobile phase (A) at 0-10 min: 95%, 10-15 min: 60%, 15-17 min: 10%, 17-20 min: 10%, 20-25 min: 95%, **Flow rate:** 0.7 ml/min, **Temp:** 30°C
**Curcumin Content. (%w/w)**	ND	**High Performance Liquid Chromatography** (SHIMADZU; Nexera LC-40 series), Inertsil ODS-4 column (3.0 mm ID x 150 mm, 5 µm), **Mobile phase:** (A) Water, (B) Acetonitrile at A:B ratio of 55:45, **Flow rate:** 0.5 ml/min, **Temp:** 40°C

Note: ND = Not detected

Conclusion and Certification

This report is valid only for the sample tested. The test report shall not be reproduced except in full, without written approval from the laboratory.

Certifying Officer: (Signature) *(Dr. Sunee Jeerapanich)* **Position:** Researcher, Nondestructive Quality Inspection Technology Laboratory **Date:** December 9, 2568 (B.E. 2568 is equivalent to A.D. 2025)
